# M-Type Strontium Hexaferrite Nanoestructures Derived from the Pechini Method as Magnetically Hard Adsorbents for Cadmium Removal in Aqueous Solution

**DOI:** 10.3390/ma19101992

**Published:** 2026-05-12

**Authors:** R. Murillo-Ortíz, María de Jesús Martínez-Carreón, A. Lobo Guerrero, R. Herrera-Rivera, Eduardo G. Pérez-Tijerina

**Affiliations:** 1 Centro de Investigación en Ciencias Físico Matemáticas, Universidad Autonoma de Nuevo Leon, Pedro de Alba S/N, Ciudad Universitaria, San Nicolas de los Garza C.P. 66455, Mexico; maria.martinezcr@uanl.edu.mx (M.d.J.M.-C.); maria.herrerarv@uanl.edu.mx (R.H.-R.); eduardo.pereztj@uanl.edu.mx (E.G.P.-T.); 2 Área Académica de Ciencias de la Tierra y Materiales, Universidad Autónoma del Estado de Hidalgo, Carretera Pachuca-Tulancingo km. 4.5, Mineral de la Reforma C.P. 42039, Mexico

**Keywords:** strontium hexaferrite, Pechini method, cadmium adsorption, water remediation

## Abstract

This study investigates the removal of Cd^2+^ ions from aqueous solutions using hard magnetic strontium hexaferrite (SrFe_12_O_19_) nanoparticles synthesized via the Pechini method, with an average particle size of 116 nm. The material was successfully obtained at a relatively low calcination temperature of 900 °C. The crystalline structure of the hexaferrite particles was investigated by X-ray diffraction, confirming SrFe_12_O_19_ crystalline structure. The powder samples were also characterized by Fourier transform infrared spectroscopy (FTIR). The morphology and size distribution were studied using scanning electron microscopy (SEM). Furthermore, the magnetic properties of strontium hexaferrite contribute significantly to adsorption and removal processes, primarily by acting as a recoverable magnetic adsorbent. The ferromagnetic material, with its high saturation magnetization and coercivity, responds rapidly to external magnets, facilitating the removal of contaminants and maintaining its magnetic characteristics even in complex chemical environments. For this purpose, its magnetic behavior was also studied using vibrating sample magnetometry (VSM). The experimental adsorption results were successfully modeled using PFO (pseudo—first—order) and PSO (pseudo—second—order) along with Freundlich and Langmuir isotherms, to fit the experimental adsorption data of the Cd(II) salt from the 0.1 and 0.2 mg samples at room temperature for two quantities of strontium hexaferrite at times ranging from 2.5 to 60 min. The results indicate that the strontium hexaferrite nanoparticles exhibited a 90% removal efficiency, which was the highest value. Additionally, the strontium hexaferrite can be magnetically recovered along with the adsorbed cadmium, representing a more efficient way to remediate water.

## 1. Introduction

The treatment of contaminated water is of critical importance given the widespread presence of toxic pollutants in aquatic systems. Conventional treatment methods are often ineffective at removing emerging contaminants, including heavy metals. Cadmium (Cd) is an extremely mobile element in the environment, raising significant environmental and health concerns due to its high toxicity [1,2,3]. Because of its high solubility and mobility, improper disposal and environmental degradation and recycling of batteries have resulted in substantial global dissemination of this toxic heavy metal, posing a long-term risk of environmental contamination and bioaccumulation in ecosystems [4]. Cadmium has persisted in drinking water sources for over 20 years, increasing its concentration and representing a significant risk to public health, microorganisms, plants, and animals [5,6,7,8,9]. Various strategies based on chemical, physical, or combined processes have been proposed to reduce cadmium levels in drinking water [10]. Magnetic nanomaterials (MNMs) have emerged as highly effective candidates for both adsorption and photocatalysis [11].

There are six types of hexaferrite: M, U, Y, Z, W and X. Of these, type-M hexaferrite is the most widely used [12]. In particular, M-type hexaferrite (MFe_12_O_19_) is a hard hexaferrite with a magnetoplumbite-like structure and P6_3_/mmc space group; the M site is occupied by divalent metal ions, like strontium (Sr), lead (Pb) or barium (Ba) [13] which stabilize the crystal structure and influence the material’s magnetic properties.

The M-type hexaferrite structure is reflected in RSR*S* notation, where the S block stands for cubic packing and the R block for hexagonal packing. In this case, (*) denotes a block that has been turned 180 degrees around the hexagonal c-axis. The M ion is situated between hexagonally arranged oxygen layers in M-type hexaferrite. Five distinct interstitial sites contain the 24Fe^3+^ ions: three octahedral (2a, 12k, and 4f2), one tetrahedral (4f1), and one trigonal bipyramidal (2b). The unit cell of MFe_12_O_19_ contains two formula units and exhibits a hexagonal crystal structure, with 12 Fe ions and 19 O ions per formula unit. The presence of strontium ions provides additional magnetic properties and stabilizes the structure [14]. Magnetic carbon nanotube compounds, magnetite (Fe_3_O_4_) and maghemite (γ-Fe_2_O_3_) have been thoroughly investigated for the removal of heavy metals like cadmium; however, their small particle size and oxidation susceptibility make it difficult to recover them from treated water [15,16,17,18,19,20,21].

Hexagonal nanomaterials, a class of MNMs, have demonstrated great potential for water treatment and are a scalable and economical solution for industrial water remediation due to their high magnetic coercivity and moderate saturation magnetization [22,23]. Due to their magnetic behavior, they can be manipulated and controlled, which makes them appropriate for applications requiring precise magnetic control. Hexaferrites are materials that are chemically stable and resistant to corrosion and deterioration. Even under harsh environmental conditions, their stability ensures consistent performance in wastewater treatment procedures. Hexaferrites’ large surface area and nanoscale morphology enhance interaction with contaminants, improving photocatalytic activity and adsorption. This enables the efficient removal of organic pollutants from wastewater. In order to add desired properties for particular applications, hexaferrites can currently be synthesized using various manufacturing techniques.

In this work, the Pechini technique was used to synthesize strontium hexaferrite as it is a versatile method that allows the synthesis of high-purity nanostructures with nanoscale dimensions, enhancing adsorption performance for cadmium (II) salt removal, while preserving its magnetic properties. These magnetic properties play a crucial role in the removal of Cd^2+^ from contaminated water by facilitating efficient magnetic separation of the adsorbent, effectively overcoming the limitations associated with the recovery of nanoscale materials. This feature improves the efficiency and sustainability of the treatment process by enabling the separation, recovery, and reuse of the material after heavy metal adsorption.

## 2. Materials and Methods

### 2.1. Synthesis of Materials

Strontium hexaferrite was synthesized using the Pechini method [24,25], which enables the synthesis of highly pure strontium hexaferrite nanoparticles at a low sintering temperature. Strontium nitrate (Sr(NO_3_)_2_, Meyer 99.00%, Vallejo, CA, USA), citric acid (C_6_H_8_O_7_, Sigma-Aldrich 99.50%, St. Louis, MO, USA), ethylene glycol (C_2_H_6_O_2_, Sigma-Aldrich 99.80%), and iron nitrate nonahydrate (Fe(NO_3_)_3_·9H_2_O, Sigma-Aldrich 98.00%) were the chemical reagents used in the synthesis.

The synthesis was carried out by weighing strontium nitrate and iron nitrate using a Sr:Fe molar ratio of 1:12 targeting a final yield of 1 g of product. A total of 0.2013 g of Sr(NO_3_)_2_ was mixed with 4.6592 g of Fe(NO_3_)_3_·9H_2_O in 20 mL of deionized water. The mixture was maintained under constant stirring at room temperature for 40 min. Subsequently, 11.15 mL of C_2_H_6_O_2_ and 9.606 g of C_6_H_8_O_7_ were added in 20 mL of deionized water. The solution was initially heated to 85 °C to evaporate excess water, then maintained at 80 °C to promote polyesterification. This process yields a homogeneous resin with uniformly distributed metal ions in the organic matrix. The polymer resin is further heated at 200 °C for 4 h to remove excess solvent and subsequently sintered in a Thermo Scientific furnace (Waltham, MA, USA) using a heating rate of 5 °C/min. The samples were then maintained at 900 °C for 5 h and subsequently cooled to room temperature within the furnace.

### 2.2. Characterization

The structural characteristics of SrFe_12_O_19_ were obtained by X-ray diffraction (XRD) using a Bruker diffractometer (model D8 Advance) (Billerica, MA, USA) with Cu-Kα radiation over a 2θ range of 20° to 80°, a scan speed of 4°/min and a step size of 0.02°. The MAUD program version 2.9995 (materials analysis using diffraction) was used to perform Rietveld refinement. The particle morphology and size distribution of SrFe_12_O_19_ particles were characterized using a Hitachi S-570 scanning electron microscope (SEM) (Tokyo, Japan) operated at 8 kV. The morphological characteristics of the as-prepared strontium hexaferrite were analyzed using ImageJ program version 1.54g to determine particle size distribution and average particle size from SEM micrographs. Infrared spectra were recorded at room temperature with a Thermo Scientific spectrometer (model Nicolet iS10) (Waltham, MA, USA) in the range of 4000–400 cm^−1^. Additionally, nitrogen physisorption measurements at −196 °C were performed with a Micromeritics TriStar II 3020 (Micromeritics Instrument Corporation, Norcross, GA, USA) to determine the specific surface area, pore-volume and size distribution. Samples were pretreated under vacuum at 250 °C for 8 h. Surface areas were calculated using the BET method, and pore diameters were obtained using the BJH method applied to the adsorption branch of the isotherm.

### 2.3. Cadmium Adsorption Experiments

The experiments to remove cadmium (II) salt were carried out at room temperature in aqueous solution. To maintain cadmium as a free divalent cation and avoid the formation of hydroxylated species that precipitate at higher pH values, a batch solution containing 62 mg/L of Cd(II) was made, and 0.1 M HCl was used to adjust the pH to 3.0. This condition also relates to the adsorbent surface’s isoelectric (or zero point) pH, where there is little electrostatic repulsion toward cations or anions, minimizing electrostatic effects and allowing other mechanisms (e.g., chelation or specific ion exchange) to be evaluated. The intrinsic affinity of the strontium hexaferrite surface under acidic conditions, where a high concentration of H+ ions creates a competitive environment for Cd(II) ions for active sites, can also be evaluated by assessing the adsorption process at pH 3.0, offering a rigorous test of the adsorbent’s efficacy [26,27].

Two different adsorbent dosages of strontium hexaferrite used as the adsorbent for Cd(II) salt were tested: 0.1 mg and 0.2 mg. In each case, 50 mL of the cadmium salt was mixed with 0.1 and 0.2 mg of adsorbents, under constant stirring. The Cd(II) adsorption by hexaferrite nanoparticles was evaluated at different time periods: 2.5, 10.0, 15.0, 30.0, and 60.0 min. After the Cd^2+^ salt absorption, the strontium hexaferrite particles were separated from the liquid phase using a permanent magnet. Residual Cd^2+^ concentration was determined by a PERSEE TAS-990 atomic absorption spectrophotometer Part of Beijing Beifen-Ruili (Beijing, China) equipped with a graphite furnace (GFAAS). Finally, the amount of cadmium adsorbed per unit of mass of strontium hexaferrite q_e_ (mg/g) was determined by Equation (1) [28]

(1) $$q_{\;e}=\frac{\left(C_{0}-C_{e}\right)\cdot V}{m}$$

While the removal efficiency η (%) is given by Equation (2) [29].

(2) $$\eta \left(\%\right)=\frac{\left(C_{0}-C_{e}\right)}{C_{0}}\cdot 100$$

where $C_{0}$ is the initial concentration, $C_{e}$ is the equilibrium concentration, V corresponds to the volume of the solution and m is the mass of the adsorbent.

## 3. Results and Discussion

**Figure 1:**
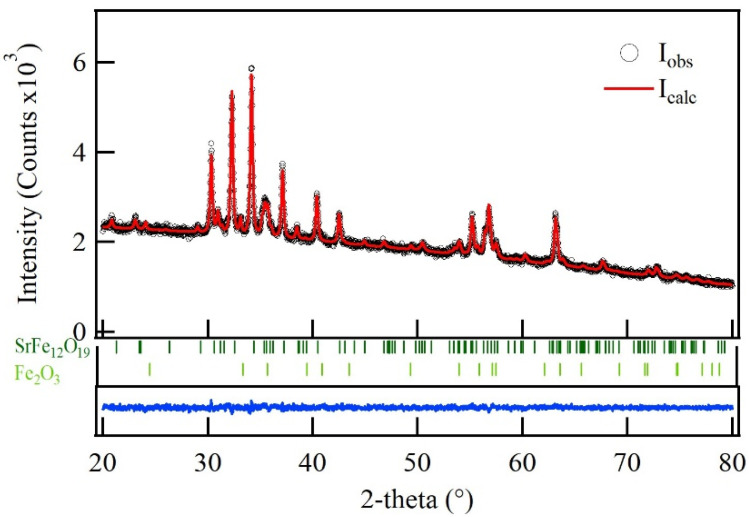
X-ray diffractogram of SrFe_12_O_19_ with the calculated Rietveld profile. At the bottom is showed the difference curve in blue (I_obs_ − I_calc_).

The X-ray diffraction (XRD) pattern of SrFe_12_O_19_ and the Rietveld refinement performed using the MAUD program are shown in Figure 1 [30]. The structural model for the Rietveld analysis was proposed using strontium hexaferrite (Crystallography Open Database code: 1006000) and iron oxide hematite (Crystallography Open Database code: 1011240) was included in the Rietveld refinement as a secondary phase, attributed to the low sintering temperature. The Rietveld fit, represented by the continuous line in Figure 1, demonstrates excellent agreement with the experimental data, confirming the reliability of the refined structural model. Furthermore, the difference curve (I_obs_ − I_calc_) at the bottom further confirms the agreement between experimental data and calculated structural parameters. Table 1 lists the structural parameters of the strontium hexaferrite and the goodness of fit obtained from the Rietveld refinement. These results show that, despite the relatively low sintering temperature, highly crystalline strontium hexaferrite was obtained. However, approximately 5 wt.% of residual hematite was detected in the sample. This impurity arises from the low calcination temperature used to maintain small particle size. This reflects a trade-off between crystalline structure, particle size, and phase purity.

**Figure 2:**
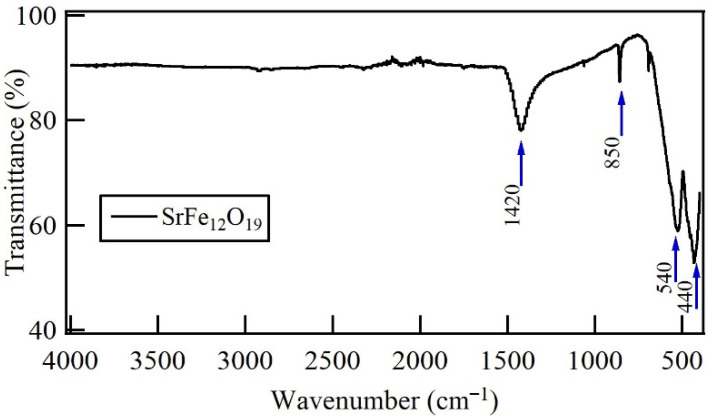
FT-IR spectrum of SrFe_12_O_19_ obtained from the sol–gel Pechini method.

FTIR spectra of SrFe_12_O_19_ submicron particles are shown in Figure 2. Here, the functional groups of these particles were identified. Bands corresponding to Fe—O—Fe vibrations at 1420 cm^−1^ are observed [31]. The metal–oxygen stretching vibrations are visible in the absorption bands at 850 cm^−1^ and below 600 cm^−1^ [32]. Specifically, the absorption bands around 850 and 540 cm^−1^ are associated with the Sr—O stretching vibration, and a band at 440 cm^−1^ corresponds to octahedral Fe—O stretching vibration [33,34,35]. The absence of additional bands confirms the crystallization of the M-type structure from the metal-nitrates [36,37].

**Figure 3:**
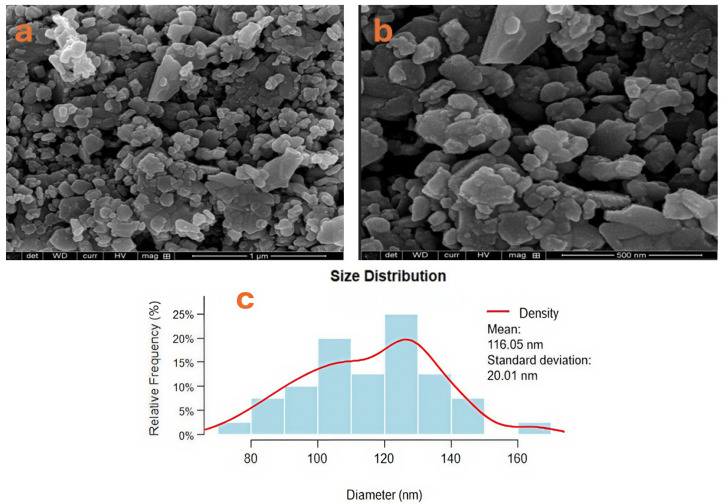
SEM images showing the morphology of the nanoparticles of the strontium hexaferrite in subsection (**a**,**b**), average grain size in subsection (**c**).

Scanning electron microscopy (SEM) was used to analyze the strontium hexaferrite particles’ morphology and size. Representative micrographs of the particle morphology and size distribution are shown in Figure 3a,b, which primarily reveal hexagonal structures. Interparticle forces and physicochemical interactions that promote particle aggregation can be attributed to the particles’ strong tendency to form agglomerates [38]. The average grain size of the synthetic material is shown in Figure 3c. Additionally, Brunauer–Emmett–Teller (BET) analysis yielded a specific surface area (SBET) of 19.86 m^2^/g, a mean pore width of 1.1198 nm, and a mean particle size of 302.1283 nm. The discrepancy between the particle size measured by SEM and the particle size estimated from BET measurements is due to particle agglomeration during the analysis.

**Figure 4:**
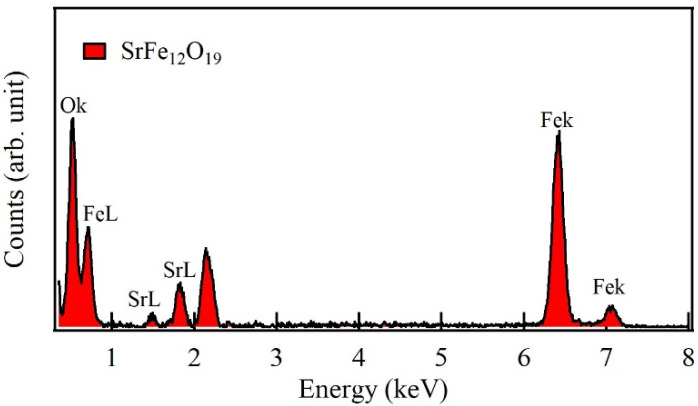
EDS spectra for the strontium hexaferrite after sintering at 900 °C for 5 h.

The elemental composition of strontium hexaferrite sub-micron particles was confirmed by EDS (energy-dispersive X-ray spectroscopy) spectra shown in Figure 4. Quantitative and theoretical elemental compositions of strontium hexaferrite are presented in Table 2. The observed deviations between theoretical and experimental compositions suggest the presence of a minor hematite phase attributed to the relatively low sintering temperature used during synthesis.

**Figure 5:**
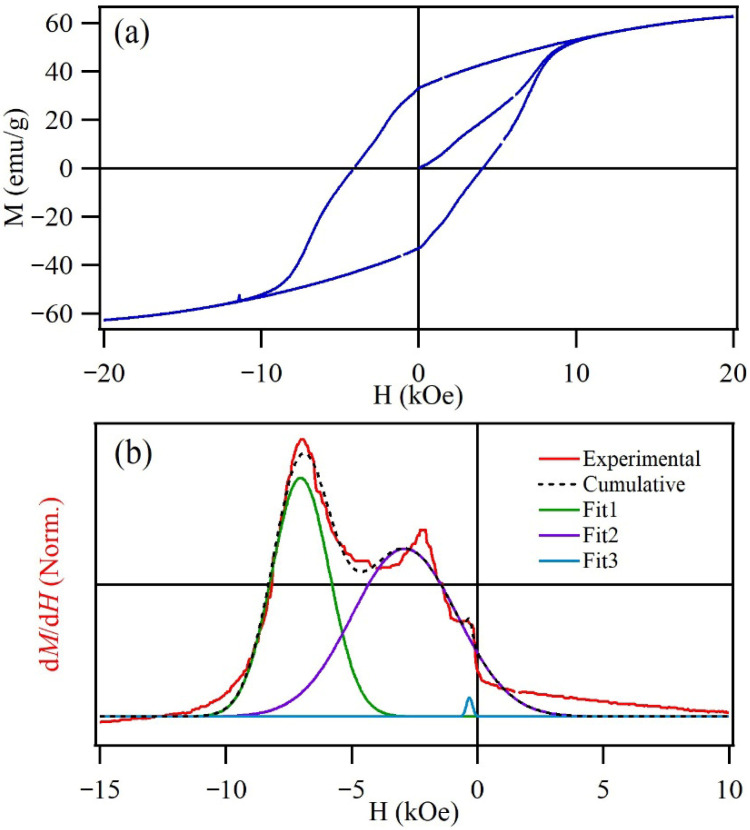
(**a**) SrFe_12_O_19_ magnetization curve derived from the Pechini method. (**b**) The dM/dH curve displaying different uncoupled magnetic contributions obtained from the upper breach’s first derivative.

The magnetization curve of the SrFe_12_O_19_ measured at room temperature is shown in Figure 5a. The maximum applied field H_max_ was 20 kOe to reach magnetic saturation (M_s_). The sample exhibited a coercive field (H_c_) equal to 6.1 kOe, remanent magnetization (M_r_) = 33.15 emu/g, M_s_ = 62.8 emu/g, and remanent squareness (M_r_/M_s_) = 0.53. These magnetic attributes are typical of a hard magnetic material, confirming the high coercivity of the material. However, the curve exhibits discontinuities revealing minor irregularities.

The first derivative of the upper branch of the magnetization curve is shown in Figure 5b to analyze the effects of different magnetic contributions. The d*M*/d*H* curve showed the presence of three distinct magnetic components. However, this behavior is mainly attributed to a broad particle size distribution, arising from particles with different magnetic behaviors (associated with their size), although all particles remain ferrimagnetic. The distinct magnetic contributions observed in the d*M*/d*H* curves may arise from varying degrees of particle agglomeration, where dipolar interactions significantly influence the magnetic behavior of the hexaferrite powder. It is important to note that these interactions are characteristic of the dry powder state used during measurement. In an aqueous medium, the particles are expected to achieve better dispersion, reducing the extent of physical agglomeration. Consequently, the robust magnetic properties evidenced by the hysteresis loops and d*M*/d*H* profiles ensure a high magnetic response of the dispersed hexaferrite particles to an external field, facilitating their efficient recovery from the treated water.

**Figure 6:**
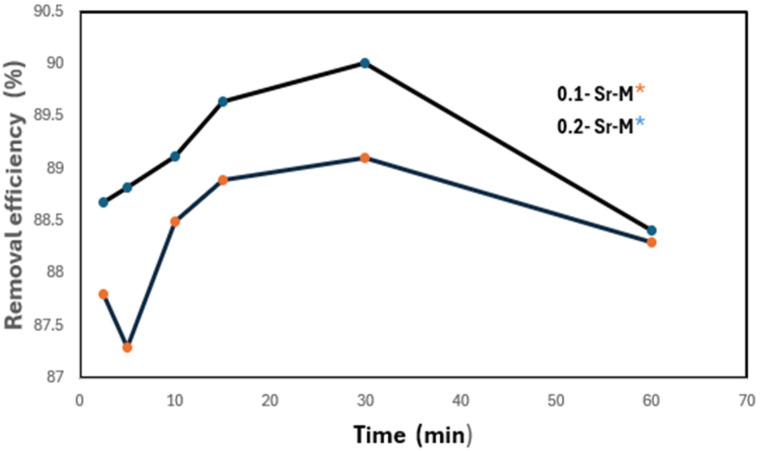
Cadmium removal efficiency, using concentrations of 0.1 and 0.2 mg of strontium hexaferrite nanoparticles-Sr-M. (The asterisks represent the color of each nanoparticle concentration).

Cd^2+^ removal experiments were conducted using strontium hexaferrite as the adsorbent at contact times of 2.5, 10, 15, 30 and 60 min. Quantitative adsorption results at different contact times are presented in Table 3. Figure 6 shows the Cd^2+^ adsorption efficiency as a function of contact time and an initial concentration of 62 mg·L^−1^ using 0.1 and 0.2 mg of SrFe_12_O_19_ nanoparticles. It was observed that Cd^2+^ adsorption occurred rapidly in the initial stage (0 to 60 min), after which the adsorption rate decreased and approached equilibrium. This behavior arises from the large number of unoccupied adsorption sites accessible to Cd^2+^ ions during the early stages of the process, and subsequently, due to repulsive interactions between adsorbed Cd^2+^ ions and reduced availability of active sites, further adsorption was hindered to occupy the remaining viable adsorption sites.

Moreover, the maximum removal efficiency depends on the adsorbent dosage. This efficiency rose as the concentration of nanoparticles increased, reaching 90% removal at 0.2 mg slightly decreasing to 88.4% at 60 min. These results indicate that Cd(II) adsorption at 0.2 mg resulted in faster adsorption kinetics than 0.1 mg, and that increasing the concentration of nanoparticles enhances the rate and extent of Cd^2+^ removal.

**Figure 7:**
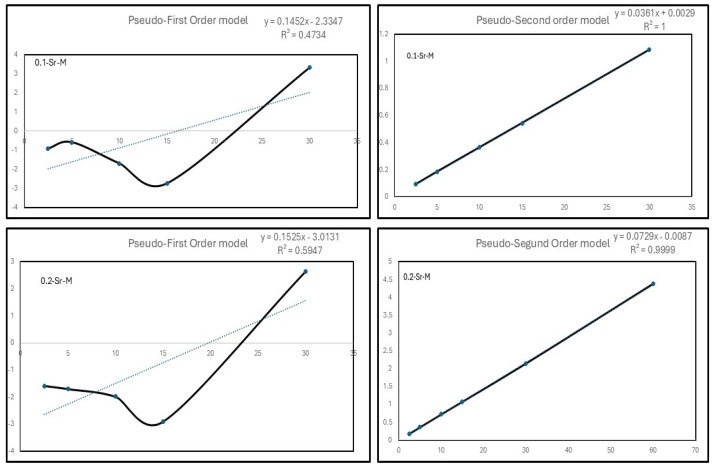
Time-dependent Cd^2+^ adsorption kinetics and removal efficiency on 0.1 Sr-M and 0.2-Sr-M.

Figure 7 presents the adsorption kinetics of Cd(II) salt, including linearized plots of the PFO (pseudo-first-order) and PSO (pseudo-second-order) kinetic models. The corresponding kinetic parameters (k_1_, k_2_, q_e_, and R^2^) for the 0.1-Sr-M and 0.2-Sr-M samples were calculated from the linearized plots and are summarized in Table 4. The PFO model exhibited poor linear correlation (R^2^ < 0.90), whereas the PSO model exhibited excellent linearity and agreement with experimental adsorption capacities (R^2^ > 0.99), with calculated adsorption capacities (q_e_,cal) closely matching experimental values (q_e_,exp) indicating that the adsorption process is well described by the PSO model and suggesting that chemisorption is the rate-limiting step. This behavior is consistent with previous reports on similar systems, such as Fe_3_O_4_-SO_3_H (pH = 7) [39], cashew nut shell resin-coated Fe_3_O_4_ nanoparticles (pH = 10) [40], sulfur-modified magnetic nanoparticles (pH = 4) [41], and Fe_3_O_4_ (pH = 3–7) [42].

The cadmium adsorption mechanism is governed by the point of zero charge (pH_pzc_) of the adsorbent, which was experimentally determined to be 2.5 for the synthesized material [20]; this justifies selecting pH 3.0 in batch experiments, as operating above the pH_pzc_ promotes deprotonation of surface hydroxyl groups (Fe–OH + OH^−^ → Fe–O^−^ + H_2_O), generating a net negative surface charge thereby enhancing electrostatic attraction of Cd^2+^ ions. Nevertheless, high removal efficiency was observed despite the presence of competing H^+^ ions indicates that the adsorption mechanism is not purely electrostatic but is predominantly governed by chemisorption, likely involving the formation of stable inner-sphere complexes through direct bonding between cadmium ions and surface oxygen atoms of the ferrite lattice, resulting in strong adsorption affinity under acidic conditions.

Two widely used kinetic models: PFO (Equation (3)) and PSO (Equation (4)), were applied to fit the Cd(II) adsorption kinetics onto MP—SF and SP—SF samples:

(3) $$\frac{dq_{t}}{dt}=k_{1}\left(q_{e}-q_{t}\right)$$

(4) $$ln\left(q_{e}-q_{t}\right)=ln\left(q_{e}\right)-k_{1}t$$

In these models, *t* (minutes) represents the contact time, while *q*_*t*_ (mg·g^−1^) and *q_e_* (mg·g^−1^) correspond to the amount of Cd^2+^ adsorbed at time *t* and at equilibrium, respectively. The rate constants for PFO and PSO models are given by *k*_1_ (min^−1^) and *k*_2_ (g·mg^−1^ min^−1^) respectively.

**Figure 8:**
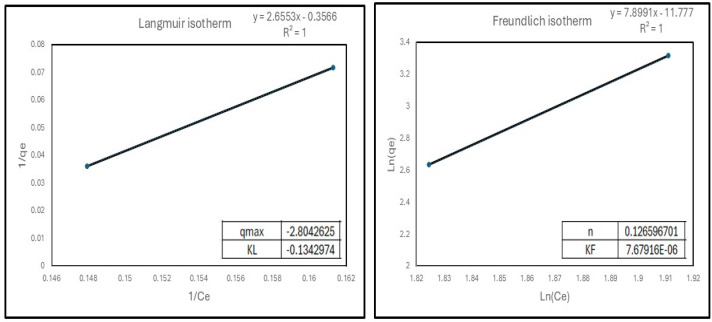
Cd(II) adsorption isotherms at initial concentrations up to 62 mg/L.

Figure 8 presents the Cd^2+^ adsorption isotherms onto MP-SF and SP-SF over an initial Cd(II) concentration range up to 62 mg/L. The MP-SF and SP-SF samples exhibited adsorption capacities of approximately 27.55 and 13.89 mg/g, respectively, without reaching saturation within the investigated concentration range, highlighting their high adsorption capacity for Cd^2+^ adsorption. The experimental data were better described by the Freundlich isotherm model, as evidenced by higher correlation coefficients (R^2^), compared to the Langmuir model. This improved fitting suggests that Cd(II) adsorption onto the 0.1-Sr-M and 0.2-Sr-M samples occurs on a heterogeneous surface, consistent with a multilayer adsorption process.

**Figure 9:**
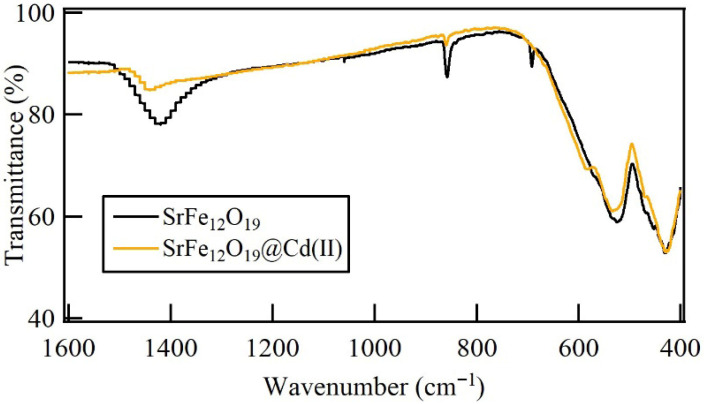
FT-IR spectrum of strontium hexaferrite obtained by the Pechini sol–gel method after cadmium absorption.

The FTIR spectra of strontium hexaferrite before and after Cd(II) adsorption are shown in Figure 9, indicating interactions between cadmium ions and the adsorbent surface. The involvement of surface functional groups in Cd(II) binding is suggested by a decrease in band intensity around 1420 cm^−1^. This behavior is explained by the displacement of protons from surface hydroxyl groups, which results in the formation of Fe–O–Cd bonds that change the local vibrational environment and reduce band intensity [43,44,45,46]. The observed adsorption behavior is attributed to the displacement of protons from surface hydroxyl groups, resulting in the formation of stable Fe–O–Cd linkages. This mechanism leads to the formation of inner-sphere complexes. In contrast to physisorption, this chemisorption process involves a direct orbital interaction between Cd^2+^ d-orbitals and oxygen p-orbitals of the ferrite surface. The robustness of this interaction is evidenced by the high removal efficiency achieved at pH 3.0, where the hexaferrite surface is only slightly above its point of zero charge pH_pzc_ = 2.5, yet remains active for metal adsorption.

Additionally, bands at 1444 and 983 cm^−1^, indicate the involvement of metal–oxygen (M–O and M–O–M) stretching vibrations, suggest that hydroxylated metal groups, such as Fe–OH, play a key role in the adsorption process. These features support a chemisorption mechanism that is mainly controlled by surface complexation and ion exchange.

Even in acidic environments, the adsorbent retains its crystalline phase and structural integrity following adsorption, as evidenced by the persistence of the distinctive Fe–O bands at 585 and 430 cm^−1^. Overall, the FT-IR data show that chemisorption processes involving surface complexation and ion exchange, which alter the local bonding environment without altering the bulk crystal structure, are primarily responsible for Cd(II) adsorption.

## 4. Conclusions

In this work, the Pechini technique was used to synthesize hexagonal SrFe_12_O_19_ nanoparticles with an average size of 116 nm for the adsorption of Cd(II) from contaminated water. High adsorption capacities and efficiencies at pH 3 and room temperature were shown by adsorption experiments carried out at various contact times.

The variation in Cd(II) adsorption efficiency indicates that the adsorption mechanism, which involves chemical interactions between the adsorbent surface and the adsorbate, is governed by chemisorption as the rate-limiting step. For the 0.1 mg and 0.2 mg Sr-M samples, respectively, maximum adsorption efficiencies of 89% and 90% were attained. The adsorption of Cd(II) onto the adsorbent surface was confirmed by changes in FTIR spectra. The results of the experiment showed that it represents a promising candidate for water treatment and that it can be efficiently recovered and reused as an adsorbent.

## Figures and Tables

**Table 1:** Structural results of the Rietveld analysis for SrFe_12_O_19_.

Phase	Purity wt (%)	Lattice Parameters		Density (g/cm^3^)	Crystallite Size (nm)	Microstrain	Crystallinity (%)	Goodness of Fit		
		a (Å)	c (Å)					R_b_	R_w_	c^2^
SrFe_12_O_19_	95 ± 3	5.8848 ± 0.0001	23.0773 ± 0.0009	5.09	72 ± 1	3.5 × 10^−7^	98.8	1.93	2.42	1.05

**Table 2:** Quantitative analysis of the energy dispersive X-ray spectroscopy.

	Sr (wt.%)	Fe (wt.%)	O (wt.%)	Total
Experimental	9.28	58.52	32.20	100.00%
Theoretical	8.25	63.12	28.63	100.00%

**Table 3:** Impact of adsorbent dosage on the final Cd^2+^ concentrations (C_f_), adsorption capacities (q_e_) and removal efficiencies (η) of the 0.1 Sr-M and 0.2-Sr-M samples.

Adsorbent Dosage (mg)	Time (min)	C_f_ (mg/L)	q_e_ (mg/g)	n (%)
0.1-Sr-M	2.5	7.56	27.22	87.80
	5.0	7.88	27.06	87.20
	10.0	7.13	27.43	88.50
	15.0	6.89	27.55	88.80
	30.0	6.76	27.62	89.00
	60.0	7.26	27.37	88.29
0.2-Sr-M	2.5	7.02	13.74	88.67
	5.0	6.93	13.76	88.82
	10.0	6.75	13.81	89.11
	15.0	6.42	13.89	89.64
	30.0	6.20	13.95	90.00
	60.0	7.19	13.70	88.40

**Table 4:** Kinetics parameters for adsorption of Cd^2+^ on the 0.1-Sr-M and 0.2 Sr-M.

Sample	Pseudo First Order Model			Pseudo Second Order Model		
	q_e_ (mg/g)	k_1_ (min^−1^)	R^2^	q_e_ (mg/g)	k_2_ g/(mg∙min)	R^2^
0.1-Sr-M	0.09668395	−0.1452	0.4734	27.700831	0.4493828	1
0.2-Sr-M	0.04991391	−0.1525	0.5947	13.986014	0.9129018	0.9999

## Data Availability

The original contributions presented in this study are included in the article. Further inquiries can be directed to the corresponding authors.
